# Supplementary material for: Rapid Whole-Exome Sequencing as a Diagnostic Tool in a Neonatal/Pediatric Intensive Care Unit
Source: J Clin Med. 2020 Jul 13;9(7):2220. doi: 10.3390/jcm9072220 (PMC7408678; doi:10.3390/jcm9072220)
Supplement: Supplementary file 1 [file jcm-09-02220-s001.pdf]

Supplementary Materials: Rapid Whole-Exome Sequencing as a Diagnostic Tool in a Neonatal/Pediatric Intensive Care Unit

Robert Śmigiel, Mateusz Biela, Krzysztof Szmyd, Michal Błoch, Elżbieta Szmida, Paweł Skiba, Anna Walczak, Piotr Gasperowicz, Joanna Kosińska, Małgorzata Rydzanicz, Piotr Stawiński, Anna Biernacka, Marzena Zielińska, Waldemar Gołębiowski, Agnieszka Jalowska, Grażyna Ohia, Bożena Głowska, Wojciech Walas, Barbara Królak-Olejnik, Paweł Krajewski, Jolanta Sykut-Cegielska, Maria M. Sąsiadek and Rafał Płoski

Table S1. Variants pathogenicity criteria.

ID/SEX	Gene/OMIM (Number, Disease, Inheritance)	Variant (s) hg38	Pathogenicity Verdict According to ACMG Classification[#] (https://varsome.com)	Explanation	Frequency in GnomAD
1/M	*SCO2* 604377 Leigh syndrome AR	compound heterozygote NM_005138.3:			
		chr22:050524395-C>CTGAGTCACTGCTGCATGCT c.16_17insAGCATGCAGCAGTGACTCA; p.(Arg6GlnfsTer82)	Pathogenic	PVS1: Null variant (frame-shift) affecting gene *SCO2*, which is a known mechanism of disease (gene has 19 known pathogenic variants which is greater than minimum of 3), associated with Myopia 6, Leigh syndrome, Cardioencephalomyopathy, fatal infantile, due to cytochrome c oxidase deficiency 1 and Hypertrophic cardiomyopathy.	0.000576

			PM2: GnomAD exomes homozygous allele count = 0 is less than 3 threshold for recessive gene *SCO2* (unable to check gnomAD exomes coverage). GnomAD genomes homozygous allele count = 0 is less than 3 threshold for recessive gene *SCO2* (good gnomAD genomes coverage = 31.5). PP5: ClinVar classifies this variant as Likely Pathogenic, rated 1 star, criteria provided, single submitter, with 2 submissions, 1 publication (PubMed: 20159436).[1]	
	chr22: 50523994-C>T c.418G>A; p.(Glu140Lys)	Pathogenic	PS3: ClinVar classifies this variant as Pathogenic, rated 2 stars backed by potential functional studies (requires user validation) mentioned in abstract of article (PubMed: 11673586) [2] PM1: UniProt protein *SCO2*_HUMAN domain 'Thioredoxin' has 12 non-VUS, non-synonymous, coding variants (8 pathogenic and 4 benign), pathogenicity = 66.7%	0.0000796

			which is more than threshold 33.3%.<br>PM2: GnomAD exomes homozygous allele count = 0 is less than 3 threshold for recessive gene *SCO2* (unable to check gnomAD exomes coverage). GnomAD genomes homozygous allele count = 0 is less than 3 threshold for recessive gene *SCO2* (good gnomAD genomes coverage = 32.4).<br>PP2: 9 out of 15 non-VUS missense variants in gene *SCO2* are pathogenic = 60.0% which is more than threshold of 51.0%, and 19 out of 58 clinically reported variants in gene *SCO2* are pathogenic = 32.8% which is more than threshold of 12.0%.<br>PP3: Pathogenic computational verdict based on 9 pathogenic predictions from DEOGEN2, EIGEN, FATHMM-MKL, M-CAP, MVP, MutationAssessor, MutationTaster, REVEL and SIFT vs 1 benign prediction from PrimateAI.

2/M	*SCO2*<br>604377<br>Leigh syndrome<br>AR	homozygote<br>NM_005138.3:<br>chr22: 50523994-C>T<br>c.418G>A; p.(Glu140Lys)	Pathogenic	As above	0.0000796
3/M	*POLG*<br>203700<br>Alpers syndrome<br>AR	homozygote<br>NM_001126131.2:<br>chr15:89320885-G>C<br>c.2862C>G; p.(Ile954Met)	Likely Pathogenic	PM1: Hot-spot of length 61 base-pairs has 11 non-VUS coding variants (11 pathogenic and 0 benign), pathogenicity = 100.0%, qualifies as hot-spot<br>PM2: Variant not found in gnomAD exomes (unable to check gnomAD exomes coverage).<br>Variant not found in gnomAD genomes (good gnomAD genomes coverage = 32.6).<br>PP2: 130 out of 190 non-VUS missense variants in gene *POLG* are pathogenic = 68.4% which is more than threshold of 51.0%, and 207 out of 970 clinically reported variants in gene *POLG* are pathogenic = 21.3% which is more than threshold of 12.0%.<br>PP3: Pathogenic computational verdict based on 8 pathogenic predictions from DEOGEN2, FATHMM-MKL, M-CAP, MVP,	0

				MutationAssessor, MutationTaster, REVEL and SIFT vs 1 benign prediction from EIGEN.	
4/F	*GBE1*<br>232500<br>Glycogen storage disease IV - perinatal severe form (Anderson syndrome)<br>AR	compound heterozygote<br>NM_005158.3:			
		Chr3:81648854-A>G c.691+2T>C; p.?	Pathogenic	PVS1: Null variant (intronic within ±2 of splice site) affecting gene *GBE1*, which is a known mechanism of disease (gene has 38 known pathogenic variants which is greater than minimum of 3), associated with Glycogen storage disease IV.<br>PP5: ClinVar classifies this variant as Pathogenic, rated 2 stars, with 11 submissions, 10 publications and no conflicts. [3–5]<br>PM2: GnomAD exomes homozygous allele count = 0 is less than 3 threshold for recessive gene *GBE1* (unable to check gnomAD exomes coverage). GnomAD genomes homozygous allele count = 0 is less than 3 threshold for recessive gene *GBE1*	0.000895

			(good gnomAD genomes coverage = 31.0).<br>PP3: Pathogenic computational verdict based on 3 pathogenic predictions from EIGEN, FATHMM-MKL and MutationTaster vs no benign predictions.	
	Chr3:81499187-C>T<br>c.1975G>A; p.(Gly659Arg)	Uncertain Significance	PM2: Variant not found in gnomAD exomes (unable to check gnomAD exomes coverage).<br>Variant not found in gnomAD genomes (good gnomAD genomes coverage = 31.4).<br>PP2: 17 out of 29 non-VUS missense variants in gene *GBE1* are pathogenic = 58.6% which is more than threshold of 51.0%, and 38 out of 149 clinically reported variants in gene *GBE1* are pathogenic = 25.5% which is more than threshold of 12.0%.<br>PP3: Pathogenic computational verdict based on 9 pathogenic predictions from DEOGEN2, EIGEN, FATHMM-MKL, M-CAP, MVP, MutationAssessor,	0

				MutationTaster, REVEL and SIFT vs 1 benign prediction from PrimateAI.	
5/F	PC<br>216150<br>Pyruvate carboxylase deficiency<br>AR	compound heterozygote<br>NM_022172.3:			
		Chr11:66866282-G>A<br>c.1090C>T; p.(Gln364Ter)	Pathogenic	PVS1: Null variant (nonsense) affecting gene *PC*, which is a known mechanism of disease (gene has 38 known pathogenic variants which is greater than minimum of 3), associated with Pyruvate carboxylase deficiency.<br>PM2: Variant not found in gnomAD exomes (unable to check gnomAD exomes coverage).<br>Variant not found in gnomAD genomes (good gnomAD genomes coverage = 31.2).<br>PP3: Pathogenic computational verdict based on 3 pathogenic predictions from EIGEN, FATHMM-MKL and MutationTaster vs no benign predictions.	This variant does not have a gnomAD genomes entry (czy pisać 0??)
		Chr11:66863920-C>G<br>c.1222G>C; p.(Asp408His)	Pathogenic	PM1: UniProt protein PYC_HUMAN domain 'Biotin carboxylation' has 16 non-VUS, non-	This variant does not have a

		synonymous, coding variants (13 pathogenic and 3 benign), pathogenicity = 81.2% which is more than threshold 33.3%.<br>PM2: Variant not found in gnomAD exomes (unable to check gnomAD exomes coverage).<br>Variant not found in gnomAD genomes (good gnomAD genomes coverage = 32.3).<br>PP2: 24 out of 35 non-VUS missense variants in gene PC are pathogenic = 68.6% which is more than threshold of 51.0%, and 38 out of 242 clinically reported variants in gene *PC* are pathogenic = 15.7% which is more than threshold of 12.0%<br>PP3: 24 out of 35 non-VUS missense variants in gene PC are pathogenic = 68.6% which is more than threshold of 51.0%, and 38 out of 242 clinically reported variants in gene *PC* are pathogenic = 15.7% which is more than threshold of 12.0%	gnomAD genomes entry

6/M	*AIFM1*<br>300816<br>Combined oxidative phosphorylation deficiency 6<br>XLR	hemizygote<br>NM_004208.3:<br>chrX:130133411-C>G<br>c.1350G>C; p.(Arg450Ser)	Pathogenic	PM1: UniProt protein AIFM1_HUMAN region of interest 'FAD-dependent oxidoreductase' has 34 non-VUS, non-synonymous, coding variants (31 pathogenic and 3 benign), pathogenicity = 91.2% which is more than threshold 33.3%.<br>PM2: Variant not found in gnomAD exomes (unable to check gnomAD exomes coverage).<br>Variant not found in gnomAD genomes (good gnomAD genomes coverage = 23.2).<br>PP2: 40 out of 50 non-VUS missense variants in gene *AIFM1* are pathogenic = 80.0% which is more than threshold of 51.0%, and 42 out of 134 clinically reported variants in gene *AIFM1* are pathogenic = 31.3% which is more than threshold of 12.0%.<br>PP3: Pathogenic computational verdict based on 6 pathogenic predictions from FATHMM-MKL, M-CAP, MVP, MutationAssessor,	0

				MutationTaster and SIFT vs 2 benign predictions from DEOGEN2 and REVEL.	
7/F	*ABCA3*<br>610921<br>Surfactant metabolism dysfunction, pulmonary, 3 (SMPD3)<br>AR	homozygote<br>NM_001089.3:<br>Chr16:002323532-C>T<br>c.604G>A; p.(Gly202Arg)	Uncertain Significance	PM2: GnomAD exomes homozygous allele count = 0 is less than 3 threshold for recessive gene *ABCA3* (unable to check gnomAD exomes coverage). GnomAD genomes homozygous allele count = 0 is less than 3 threshold for recessive gene *ABCA3* (good gnomAD genomes coverage = 31.2).<br>PP3: Pathogenic computational verdict based on 9 pathogenic predictions from DEOGEN2, EIGEN, FATHMM-MKL, M-CAP, MVP, MutationAssessor, MutationTaster, REVEL and SIFT vs 1 benign prediction from PrimateAI.	0.0000159
8/F	*MAGEL2*<br>615547<br>Schaaf-Yang syndrome<br>AD	*de novo*<br>NM_019066.4:<br>Chr15:23644849-C>T<br>c.2894G>A; p.(Trp965Ter)	Pathogenic	PVS1: Null variant (nonsense) affecting gene MAGEL2, which is a known mechanism of disease (gene has 73 known pathogenic variants which is greater than minimum of 3), associated with Schaaf-	0

				Yang syndrome (Prader-Willi-like syndrome).<br>PM2: Variant not found in gnomAD exomes (unable to check gnomAD exomes coverage).<br>Variant not found in gnomAD genomes (good gnomAD genomes coverage = 31.6).<br>PP3: Pathogenic computational verdict based on 3 pathogenic predictions from EIGEN, FATHMM-MKL and MutationTaster vs no benign predictions.	
9/F	*NALCN*<br>611549<br>Hypotonia, infantile, with psychomotor retardation and characteristic facies 1 (IHPRF1)<br>AR	compound heterozygote<br>NM_052867.2:			
		chr13:101111216-G>A<br>c.2203C>T; p.(Arg735Ter)	Pathogenic	PVS1: Null variant (nonsense) affecting gene *NALCN*, which is a known mechanism of disease (gene has 77 known pathogenic variants which is greater than minimum of 3), associated with Congenital contractures of the limbs and face, hypotonia, and developmental delay and Hypotonia, infantile, with psychomotor retardation and characteristic facies 1.	0.00000807

				PM2: GnomAD exomes homozygous allele count = 0 is less than 3 threshold for recessive gene *NALCN* (unable to check gnomAD exomes coverage). GnomAD genomes homozygous allele count = 0 is less than 3 threshold for recessive gene *NALCN* (good gnomAD genomes coverage = 31.4). PP3: Pathogenic computational verdict based on 3 pathogenic predictions from EIGEN, FATHMM-MKL and MutationTaster vs no benign predictions. PP5: ClinVar classifies this variant as Pathogenic, rated 1 star, criteria provided, single submitter, with 1 submission.	
		Chr13:101229388-C>A c.1626+5G>T; p-	Uncertain Significance (note: Predicted to strongly affect splicing -ADA Score 0.9997. If this is taken into account the verdict is „pathogenic”).	PM2: Variant not found in gnomAD exomes (unable to check gnomAD exomes coverage). Variant not found in gnomAD genomes (good gnomAD genomes coverage = 32.0).	0?

10/F	*ACTA1*<br>161800<br>Nemaline myopathy<br>AD	*de novo*<br>NM_001100.3:<br>Chr1:229432567-C>A<br>c.443G>T, p.(Gly148Val)	Likely Pathogenic	PM1: Hot-spot of length 61 base-pairs has 13 non-VUS coding variants (13 pathogenic and 0 benign), pathogenicity = 100.0%, qualifies as hot-spot.<br>PM2: Variant not found in gnomAD exomes (unable to check gnomAD exomes coverage).<br>Variant not found in gnomAD genomes (good gnomAD genomes coverage = 30.5).<br>PM5: Alternative variant chr1:229432567 C⇒T (Gly148Asp) is classified Pathogenic by UniProt Variants (and confirmed using ACMG).<br>Alternative variant chr1:229432568 C⇒ G (Gly148Arg) is classified Pathogenic, 1 star, by ClinVar (and confirmed using ACMG).<br>Alternative variant chr1:229432568 C⇒ T (Gly148Ser) is classified Likely Pathogenic, 2 stars, by ClinVar (and confirmed using ACMG).<br>PP2: 168 out of 168 non-VUS missense variants in	0

				gene ACTA1 are pathogenic = 100.0% which is more than threshold of 51.0%, and 186 out of 284 clinically reported variants in gene *ACTA1* are pathogenic = 65.5% which is more than threshold of 12.0%<br>PP3: Pathogenic computational verdict based on 9 pathogenic predictions from DEOGEN2, EIGEN, FATHMM-MKL, M-CAP, MVP, MutationAssessor, MutationTaster, PrimateAI and REVEL vs no benign predictions.	
12/M	*TRMT10C*<br>616974<br>Mitochondrial disease (Combined oxidative phosphorylation deficiency 30)<br>AR	compound heterozygote<br>NM_017819.4:			
		Chr3:101565509-T>C<br>c.728T>C; p.(Ile243Thr)	Uncertain Significance	PM1: UniProt protein TM10C_HUMAN domain 'SAM-dependent MTase TRM10-type' has 1 non-VUS, non-synonymous, coding variant (1 pathogenic and 0 benign), pathogenicity = 100.0% which is more than threshold 33.3%.<br>PM2: GnomAD exomes homozygous allele count = 0 is less than 3 threshold	0.00000401

				for recessive gene TRMT10C (unable to check gnomAD exomes coverage).<br>Variant not found in gnomAD genomes (good gnomAD genomes coverage = 31.3).<br>PP3: Pathogenic computational verdict based on 5 pathogenic predictions from EIGEN, FATHMM-MKL, MutationTaster, PrimateAI and SIFT vs 4 benign predictions from DEOGEN2, M-CAP, MVP and REVEL	
		Chr3:101565164-C>CA<br>c.393_3394insA; p.(Tyr132IlefsTer15)	Likely Pathogenic	PVS1: Frameshift variant<br>PM2: GnomAD genomes homozygous allele count = 0 is less than 3 threshold for recessive gene *TRMT10C* Note: GnomAD have indicated that the data quality is suspect.	0.00177<br>Note: GnomAD have indicated that the data quality is suspect.
13/F	*NFASC*<br>618356<br>New disorder described (Neurodevelopmenta l disorder with central and	homozygote<br>NM_015090.3:<br>Chr1:204984059-C>T<br>c.2491C>T; p.(Arg831Ter)	Pathogenic	PVS1: Null variant (nonsense) affecting gene *NFASC*, which is a known mechanism of disease (gene has 5 known pathogenic variants which is greater than minimum of	0.00000795

peripheral motor dysfunction)<br>AR		3), associated with Neurodevelopmental disorder with central and peripheral motor dysfunction.<br>PM2: GnomAD exomes homozygous allele count = 0 is less than 3 threshold for recessive gene *NFASC* (unable to check gnomAD exomes coverage). GnomAD genomes homozygous allele count = 0 is less than 3 threshold for recessive gene *NFASC* (good gnomAD genomes coverage = 31.0).<br>PP5: ClinVar classifies this variant as Pathogenic, rated 0 stars, no assertion criteria provided, with 1 submission, 1 publication (PubMed: 30124836) [6]. Using strength Moderate because VarSome users have linked 1 article: 30124836, stating this variant is pathogenic.<br>PP3: Pathogenic computational verdict based on 2 pathogenic predictions from FATHMM-MKL and

				MutationTaster vs 1 benign prediction from EIGEN.	
14/M	*NARS1*<br>108410<br>A new disease suspected<br>AR	homozygote<br>NM_004539.4:<br>Chr18:57606713-A>G<br>c.1040T>C; p.(Phe347Ser)	Uncertain Significance	PM2: Variant not found in gnomAD exomes (unable to check gnomAD exomes coverage).<br>Variant not found in gnomAD genomes (good gnomAD genomes coverage = 30.5).<br>PP3: Pathogenic computational verdict based on 9 pathogenic predictions from DEOGEN2, EIGEN, FATHMM-MKL, M-CAP, MVP, MutationAssessor, MutationTaster, REVEL and SIFT vs 1 benign prediction from PrimateAI.	0
15/M	*DCAF5*<br>603812<br>A new disease suspected AD	*de novo*<br>NM_003861.3:<br>14:069055385-G>C<br>c.1301C>G; p.(Ser434Ter)	Pathogenic	PVS1: Null variant (nonsense) affecting gene DCAF5, which is a known mechanism of disease (the ExAC probability LOF intolerant (pLI) = 1 is greater than 0.7 threshold and probability LOF tolerant (pNull) = 0 is less than 0.3 threshold).<br>PM2: Variant not found in gnomAD exomes (unable to check gnomAD exomes coverage).	0

				Variant not found in gnomAD genomes (good gnomAD genomes coverage = 31.3).<br>PP3: Pathogenic computational verdict based on 3 pathogenic predictions from EIGEN, FATHMM-MKL and MutationTaster vs no benign predictions.	
18/M	*SCO2*<br>604377<br>Leigh syndrome<br>AR	homozygote<br>NM_005138.3:<br>chr22: 50523994-C>T<br>c.418G>A; p.(Glu140Lys)	Pathogenic	AS IN PATIENT 1 AND 2	0.0000796

F—female, M—male

References

1. Joost, K.; Rodenburg, R.; Piirsoo, A.; van den Heuvel, B.; Zordania, R.; Ounap, K. A novel mutation in the SCO2 gene in a neonate with early-onset cardioencephalomyopathy. *Pediatr. Neurol.* **2010**, *42*, 227–230, doi:10.1016/j.pediatrneurol.2009.10.004.
2. Jaksch, M.; Horvath, R.; Horn, N.; Auer, D.P.; Macmillan, C.; Peters, J.; Gerbitz, K.D.; Kraegeloh-Mann, I.; Muntau, A.; Karcagi, V.; et al. Homozygosity (E140K) in SCO2 causes delayed infantile onset of cardiomyopathy and neuropathy. *Neurology* **2001**, *57*, 1440–1446, doi:10.1212/wnl.57.8.1440.
3. Ravenscroft, G.; Thompson, E.M.; Todd, E.J.; Yau, K.S.; Kresoje, N.; Sivadorai, P.; Friend, K.; Riley, K.; Manton, N.D.; Blumbergs, P.; et al. Whole exome sequencing in foetal akinesia expands the genotype-phenotype spectrum of GBE1 glycogen storage disease mutations. *Neuromuscul. Disord.* **2013**, *23*, 165–169, doi:10.1016/j.nmd.2012.11.005.
4. Bendroth-Asmussen, L.; Aksglaede, L.; Gernow, A.B.; Lund, A.M. Glycogen Storage Disease Type IV: A Case With Histopathologic Findings in First-Trimester Placental Tissue. *Int. J. Gynecol. Pathol.* **2016**, *35*, 38–40, doi:10.1097/PGP.0000000000000214.
5. Dainese, L.; Adam, N.; Boudjemaa, S.; Hadid, K.; Rosenblatt, J.; Jouannic, J.-M.; Heron, D.; Froissart, R.; Coulomb, A. Glycogen Storage Disease Type IV and Early Implantation Defect: Early Trophoblastic Involvement Associated with a New GBE1 Mutation. *Pediatr. Dev. Pathol.* **2016**, *19*, 512–515, doi:10.2350/14-09-1557-CR.1.

6. Smigiel, R.; Sherman, D.L.; Rydzanicz, M.; Walczak, A.; Mikolajkow, D.; Krolak-Olejnik, B.; Kosinska, J.; Gasperowicz, P.; Biernacka, A.; Stawinski, P.; et al. Homozygous mutation in the Neurofascin gene affecting the glial isoform of Neurofascin causes severe neurodevelopment disorder with hypotonia, amimia and areflexia. *Hum. Mol. Genet.* **2018**, *27*, 3669–3674, doi:10.1093/hmg/ddy277.

© 2020 by the authors. Licensee MDPI, Basel, Switzerland. This article is an open access article distributed under the terms and conditions of the Creative Commons Attribution (CC BY) license (http://creativecommons.org/licenses/by/4.0/).
